# Enhancement of chondrogenesis of adipose-derived stem cells in HA-PNIPAAm-CL hydrogel for cartilage regeneration in rabbits

**DOI:** 10.1038/s41598-018-28893-x

**Published:** 2018-07-12

**Authors:** Chau-Zen Wang, Rajalakshmanan Eswaramoorthy, Tzu-Hsiang Lin, Chung-Hwan Chen, Yin-Chih Fu, Chih-Kuang Wang, Shun-Cheng Wu, Gwo-Jaw Wang, Je-Ken Chang, Mei-Ling Ho

**Affiliations:** 1 Graduate Institute of Medicine, College of Medicine, Kaohsiung Medical University, Kaohsiung, Taiwan; 2 Orthopaedic Research Center, Kaohsiung Medical University, Kaohsiung, Taiwan; 3 Department of Physiology, College of Medicine, Kaohsiung Medical University, Kaohsiung, Taiwan; 4 Department of Orthopedics, College of Medicine, Kaohsiung Medical University, Kaohsiung, Taiwan; 5 Graduate Institute of Medicine, Kaohsiung Medical University, Kaohsiung, Taiwan; 6 Department of Medicinal and Applied Chemistry, Kaohsiung Medical University, Kaohsiung, Taiwan; 7 Department of Orthopaedic Surgery, University of Virginia, Charlottesville, VA USA; 8 Department of Orthopaedics, Kaohsiung Municipal Ta-Tung Hospital, Kaohsiung, Taiwan; 9 Division of Adult Reconstruction Surgery, Department of Orthopedics, Kaohsiung Medical University Hospital, Kaohsiung Medical University, Kaohsiung, Taiwan; 10 Department of Marine Biotechnology and Resources, National Sun Yat-sen University, Kaohsiung, Taiwan; 11 Department of Medical Research, Kaohsiung Medical University Chung Ho Memorial Hospital, Kaohsiung, Taiwan; 12 Department of Applied Chemistry, Adama Science and Technology University, Adama, Ethiopia

## Abstract

Injectable thermoresponsive hydrogels have the advantages of effective cell delivery and minimal invasion for tissue engineering applications. In this study, we investigated the chondroinductive potential of newly developed hyaluronic acid (HA)-modified thermoresponsive poly(N-isopropylacrylamide) (HA-PNIPAAm-CL) hydrogels on enhancing rabbit ADSC (rADSC) chondrogenesis *in vitro* and in the synovial cavity of rabbit. The HA-mixed PNIPAAm (HA-PNIPAAm-CP) and HA-cross-linked PNIPAAm (HA-PNIPAAm-CL) were fabricated using physical interaction and chemical cross-linking methods, respectively. The *in vitro* results showed that, compared to unmodified PNIPAAm, both HA-modified hydrogels significantly increased cell viability, chondrogenic marker gene (aggrecan and type II collagen) expression and sulfide glycosaminoglycan (sGAG) formation in embedded rADSCs. However, HA-PNIPAAm-CL showed the highest rADSC viability and chondrogenesis. The chondrogenic effects of HA-modified hydrogels on rADSCs were confirmed *in vivo* by the intraarticular injection of hydrogel-embedded rADSC constructs into rabbit synovial cavities for 3 weeks and tracing with CM-DiI labeling. Neocartilage formation in the hydrogels was determined by histomorphological staining of GAG and type II collagen. *In vivo* injected rADSC/HA-PNIPAAm-CL constructs showed more hyaline cartilage formation than that of rADSC/HA-PNIPAAm-CP and rADSC/PNIPAAm constructs in the synovial cavity of rabbit. These results suggest that the HA-PNIPAAm-CL provides a suitable microenvironment to enhance ADSC chondrogenesis for articular cartilage tissue engineering applications.

## Introduction

Articular cartilage lesions often result in progressive deterioration and eventual osteoarthritis^1^. The current clinical treatment strategies face difficulty in restoring the native structure of the cartilage^2^. Tissue engineering has been suggested to provide more advantages over the present clinical strategies^3^. Tissue engineering primarily consists of three major components: cells, biomaterials and environmental factors. Adipose-derived stem cells (ADSCs) have been proposed as a potent stem cell source for articular cartilage tissue engineering because of their multi-lineage differentiation potential, ease of harvesting for autologous stem cell transplantation and high proliferative rates for *ex vivo* expansion compared with bone marrow-derived stem cells^4–6^. Poly(N-isopropylacrylamide) (PNIPAAm) is a physically cross-linked thermoresponsive hydrogel that exhibits a lower critical solution temperature (LCST) of approximately 32 °C to 37 °C in aqueous solution; the hydrogel swells below the LCST and shrinks above the LCST in water^7^. The PNIPAAm hydrogel is a non-cytotoxic, injectable liquid biomaterial that easily carries cells, fills defects at room temperature and shifts to a solid phase at physiological temperature^7^. Therefore, this hydrogel can be a suitable cell carrier for stem cell-based tissue engineering. However, PNIPAAm alone has no chondro-inductive effect on ADSCs *in vitro*. Recently, a novel approach that combines biopolymers with stimuli-responsive materials has emerged in tissue engineering to develop a “smart hydrogel”^8,9^. Among these hydrogels, thermoresponsive combined biopolymers, such as hyaluronan (HA), have received considerable interest because the resulting materials exhibit thermosensitive characteristics with necessary biological properties, including good biocompatibility, biodegradability, and/or the differentiation induction of stem cells^10^. Although a range of PNIPAAm-grafted hydrogels has been reported^11–15^, the *in vivo* effect of these hydrogels on ADSCs was poorly evaluated. In this study, we developed a new two-step copolymerization method to synthesize the HA-PNIPAAm-CL and evaluated its chondroinductive property on rADSCs *in vivo*.

Among the biopolymers, HA is the key glycosaminoglycan in the mesenchyme during the early stage of chondrogenic differentiation^5^. Notably, HA holds key physiological roles in cartilage biomechanics and is ample in the synovial fluid. HA contributes to the high viscosity and lubricating properties of the synovial fluid. Our previous study showed that HA, as a microenvironmental factor, can both initiate and enhance cell aggregation and the chondrogenesis of ADSCs and subsequently facilitate hyaline cartilaginous matrix synthesis^5^. We therefore hypothesized that an HA-modified PNIPAAm hydrogel may improve the cell viability of ADSCs and enhance ADSC chondrogenesis for articular cartilage tissue engineering. In this study, we developed two biomaterials, HA-PNIPAAm-CP and HA-PNIPAAm-CL, and investigated their efficacy on enhancing rADSC chondrogenesis for articular cartilage tissue engineering. The *in vitro* and *in vivo* effects of HA-PNIPAAm-CP and HA-PNIPAAm-CL on the viability of rADSCs, chondrogenic differentiation and hyaline cartilage matrix formation in rabbit knee synovial cavities through minimally invasive intraarticular injection methods were investigated.

## Materials and Methods

### Data availability statement

All materials, data and associated protocols are promptly available to readers without undue qualifications in material transfer agreements.

### Materials

N-isopropyl acrylamide (NIPAM) was purchased from Sigma-Aldrich (St. Louis, MO). High-molecular-weight HA was procured from Kikkoman (Japan). Dulbecco’s Modified Eagle’s Medium (DMEM), Fetal bovine serum (FBS), and antibiotics were purchased from Gibco BRL (Gaithersburg, MD).

### Isolation and culturing of rabbit adipose-derived stem cells (rADSCs)

The rADSCs were isolated from 3-month-old New Zealand white rabbit (National Laboratory Center, Taipei, Taiwan) subcutaneous adipose tissues following previously described methods^5,16,17^ with the approval of the Kaohsiung Medical University Animal Care and Use Committee, and all methods were performed in accordance with the relevant guidelines and regulations. Briefly, the isolated rADSCs were cultured and expanded at 37 °C under 5% CO_2_ in selective K-NAC medium which containing Keratinocyte-SFM (Gibco BRL, Rockville, MD), EGF-BPE (Gibco BRL, Rockville, MD), N-acetyl-L-cysteine, L-ascorbic acid 2-phosphate sequimagnesium salt (Sigma, St. Louis, MO) and 5% FBS. This medium can maintain the characterization of pluripotent stem cells and self-renewal properties of ADSCs^5,16,17^.

### Fabrication of thermoresponsive HA-PNIPAAm hydrogels

To fabricate the PNIPAAm hydrogel, 500 mg of NIPAM was dissolved in 10 mL of distilled water and purged with nitrogen for approximately 20 min at room temperature. Then, 100 µL of tetramethylethylenediamine (TEMED) and 100 µL of ammonium persulfate were added using a syringe. The polymerizing mixture was maintained below 0 °C overnight and wrapped with silver foil to protect it from light. This process was followed by a vigorous dialysis for three days to remove the unreacted starting materials. The samples were then lyophilized. The lyophilized PNIPAAm was stored at 4 °C until use.

To fabricate the HA-PNIPAAm-CP hydrogel, lyophilized PNIPAAm mixed with a 1:5 (PNIPAAm:HA) weight ratio of HA (molecular weight: 2 million Da) was dissolved in distilled water and then lyophilized. The lyophilized HA-PNIPAAm-CP was stored at 4 °C until use.

**Figure 1:**
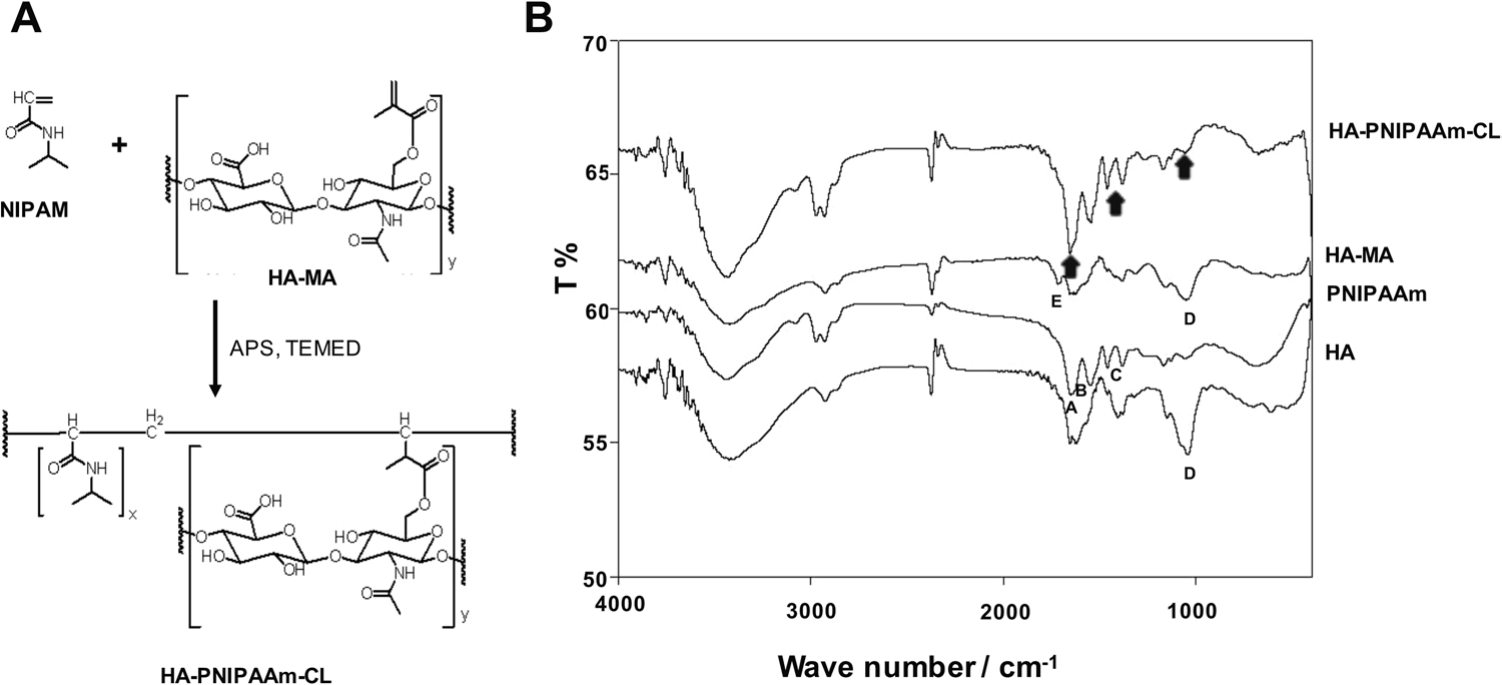
**(A**) The fabrication of HA-PNIPAAm-CL hydrogels and (**B**) the Fourier transform infrared absorption spectra of HA, PNIPAAm, HA-MA and HA-PNIPAAm-CL to confirm the functional group changes and presences of HA and PNIPAAm in cross-linked HA-PNIPAAm-CL (arrow).

The fabrication of the HA-PNIPAAm-CL hydrogel (Fig. 1A) is a two-step process. The first step is the synthesis of methacrylated hyaluronic acid (HA-MA) following a reported procedure^18^. The second step is the copolymerization of the synthesized HA-MA with NIPAM. Briefly, 500 mg of NIPAM was dissolved in 10 mLof distilled water, followed by the addition of a 1:5 (NIPAM: HA-MA) weight ratio of HA-MA. After being purged with nitrogen, 100 µL of TEMED and 100 µL of ammonium persulfate were added using a syringe. The polymerizing mixture was maintained below 0 °C overnight and wrapped with silver foil to protect the samples from light. The formed HA-PNIPAAm-CL was subjected to vigorous dialysis for 3 days to remove any unreacted starting materials, and the samples were then lyophilized. The lyophilized HA-PNIPAAm-CL was stored at 4 °C until use. The HA-PNIPAAm-CL has the same amount of HA as HA-PNIPAAm-CP after polymerization.

### Fourier transform infrared (FTIR) analysis

Fourier transform infrared (FTIR) spectroscopic analysis was performed using the KBr pellet method on a Bio-Rad infrared spectrometer (model FTS-40, Cambridge, MA). The expected pendant functionalities of a HA-PNIPAAm-CL hydrogel were confirmed by the FTIR spectrum.

### SEM examination of the microstructure inside HA-PNIPAAm hydrogels

The morphological characteristics of PNIPAAm, HA-PNIPAAm-CP and HA-PNIPAAm-CL hydrogels were observed using scanning electron microscopy (SEM, JEOL, Tokyo, Japan) after gelation. The cross-sections of freeze-dried samples were coated with gold via a sputter-coater at ambient temperature. Micrographs of all scaffolds were taken at 100X.

### Swelling ratio and shrinkage ratio (%) of HA-PNIPAAm hydrogels

Swelling/shrinkage studies were performed to calculate the water content (%) of the PNIPAAm, HA-PNIPAAm-CP and HA-PNIPAAm-CL hydrogel scaffolds, wherein 10 mg of freeze-dried hydrogels were placed in 200 µl of PBS at 37 °C. After 24 hours, these hydrogels were removed from the PBS, dabbed with a Kimwipe to remove any excess water on the surface, weighed and placed back into the buffer. The swelling ratio and shrinkage ratio (%) were calculated using the following equations. All experiments were performed 6 times^14,19^.

1 $${\rm{Swelling}}\,{\rm{ratio}}\,({\rm{SR}})=(({{\rm{W}}}_{{\rm{w}}}-{{\rm{W}}}_{{\rm{0}}})/{{\rm{W}}}_{0})\times \mathrm{100} \% $$

W_0_ and W_w_ are the initial dry weight and the wet weight, respectively.

2 $${\rm{Shrinkage}}\,{\rm{Ratio}}=(({{\rm{W}}}_{1}-{{\rm{W}}}_{2})/{{\rm{W}}}_{2})\times 100 \% $$

W_1_ is the weight of the liquid hydrogels (before gelling), and W_2_ is the weight of the solid hydrogels (after gelling).

### *In vitro* cultured rADSCs in HA-PNIPAAm hydrogels

rADSCs/hydrogel constructs composed of rADSCs and thermoresponsive hydrogels were prepared by suspending 1 × 10^6^ cells/mL rADSCs in a 5% w/v PBS solution of PNIPAAm, HA-PNIPAAm-CP and HA-PNIPAAm-CL hydrogels at 4 °C. A 200 μL aliquot of rADSCs/hydrogel was added into each 24-well cell culture plate and maintained at 37 °C for 5 min to form the gel. After gelation, rADSC/hydrogel constructs were cultured with 1 ml of basal medium containing DMEM, 10% FBS (HyClone, Logan, UT), 1% nonessential amino acids and 100 U/ml penicillin/streptomycin (Gibco-BRL, Grand Island, NY), and the culture medium was changed.

### Hydrogelation and *in vitro* degradation of HA-PNIPAAm hydrogels

For gelation time measurements, three different concentrations (2%, 5% and 10% w/v) of HA-PNIPAAm hydrogels were taken for hydrogelation (in PBS), and the gelation was analyzed from 25 °C to 40 °C by increasing the temperature 1 °C/min. For the *in vitro* degradation analysis, 200 μL of 5% w/v PBS solution of PNIPAAm, HA-PNIPAAm-CP and HA-PNIPAAm-CL hydrogel were maintained at 37 °C for 5 min to form the gel. After gelation, to mimic the *in vivo* microenvironment, hydrogel constructs were incubated in 1 ml of PBS containing hyaluronidase (100 U/mL)^20^. The samples were collected every 6 hours, and the weight loss of the hydrogels was estimated.

### Detection the viability of rADSCs in hydrogels *in vitro* using live and dead staining and MTS viability assays

To evaluate the rADSC viability and cytotoxicity after culturing in 3D hydrogels *in vitro*, rADSC/hydrogel constructs cultured in basal medium for 1 and 5 days were assessed using a live and dead cytotoxicity kit (Molecular Probes, Eugene, OR) containing calcein-AM (live dye, green) and ethidium homodimer-1 (dead dye, red). Briefly, rADSCs were isolated from the rADSC/hydrogel constructs by dissolving in PBS at 25 °C, and the cells were then collected through centrifugation. The collected cells were incubated in 1 ml of live- and dead-dye solution with 0.5 μL of calcein-AM and 2 μL of ethidium homodimer-1 in 1 ml of the standard medium for 30 min. Bright field and live/dead images of rADSC were taken using fluorescence microscopy (Eclipse 50i; Nikon Inc., MI, USA). For 3-(4,5-dimethylthiazol-2-yl)-2,5-diphenyltetrazolium bromide (MTS) viability assays (CellTiter 96® AQueous One Solution Cell Assay Promega, Madison, WI), rADSCs were isolated from the rADSC/hydrogel constructs by dissolving in PBS at 25 °C. The mitochondria activities of the rADSC cultured in hydrogels were detected by the conversion of MTS to formazan. The release of formazan product into the medium was directly proportional to the number of living cells in culture and was measured based on the absorbance at 490 nm. At the indicated time interval, the MTS reaction mixture diluted in a standard medium at a 1:5 (MTS: medium) volume ratio was added to the wells containing the cell/hydrogel constructs and then incubated at 37 °C under 5% CO_2_. After 4 h incubation, 100 μL per well of the converted MTS medium was transferred to 96-well plates, and the absorbance was measured with a microplate reader (PathTech) at 490 nm using KC junior software.

### Detecting the sGAG expression in rADSCs/hydrogel constructs *in vitro* by using Alcian blue staining and DMMB assay

Alcian blue staining was performed to detect cartilaginous matrix sulfated glycosaminoglycan (sGAG) production in the rADSC/hydrogel constructs. For Alcian blue staining, the rADSC/hydrogel constructs were cultured in 24-well plates for 5 and 7 days in basal medium. After culturing for 5 and 7 days, sGAG formation in the cultures was isolated from the rADSC/hydrogel constructs by dissolving in PBS at 25 °C and then was collected through centrifugation. The collected sGAG was fixed using 4% paraformaldehyde and stained with 0.5% Alcian blue at pH 1.0 overnight. After being washed twice with double-distilled water, the sGAG was dyed blue. A dimethylmethylene blue (DMMB) assay^21^ was used to quantify the sGAG content with shark cartilage chondroitin sulfate (Sigma-Aldrich, St. Louis, MO) as the standard. At the indicated time interval, rADSC/hydrogel constructs were lyophilized for 24 h and then digested in a papain solution (125 µg/ml papain type III, 10 mM L-cysteine, 100 mM phosphate and 10 mM EDTA at pH 6.3) for 15 h at 60 °C. A Hoechst 33258 assay^22^ was used to measure the DNA content with calf thymus DNA as the standard. The sGAG content was normalized by the DNA content.

### Detecting the chondrogenic marker gene expression in rADSC/hydrogel constructs *in vitro* using quantitative real-time PCR assay

We investigated the chondrogenic effect of PNIPAAm, HA-PNIPAAm-CP and HA-PNIPAAm-CL hydrogels on the mRNA expression of chondrogenic marker genes in rADSCs using a quantitative real-time PCR assay. The rADSC/hydrogel constructs were cultured in basal medium for 7 days. At the indicated time intervals, rADSCs were isolated from the rADSC/hydrogel constructs by dissolving the constructs in PBS at 25 °C, and then the cells were collected by centrifugation. Total RNA, isolated using the Trizol reagent, was reverse transcribed into cDNA using oligo (dT) primers and the Moloney murine leukemia virus reverse transcriptase. Quantitative real-time PCR reactions were performed in a 25-µl mixture containing cDNA, specific primers for each gene and iQ^TM^ SYBR Green Supermix using the Bio-Rad iQ5 real-time PCR detection system (Bio-Rad Laboratories, Inc., Hercules, CA). The specific PCR products were detected by the fluorescence of SYBR Green, a double-stranded DNA binding dye^23^. Dissociation (melting) curves were generated to check the specificity of each PCR reaction after the PCR reactions. The relative mRNA expression levels were calculated from the threshold cycle (*C*_t_) value of each PCR product and normalized with that of glyceraldehyde-3-phosphate dehydrogenase (GAPDH) using the comparative *C*_t_ method^24^. All experiments were performed in triplicate and repeated at least three times. The primer sequences are shown in Table 1.

**Table 1:** Sequences of primers used in the real-time PCR. Forward (F) and reverse (R) primers are shown.

Gene	PCR primers Sequence
Type II collagen (Rabbit)	F: 5′-GAC CCC ATG CAG TAC ATG CG-3′ R: 5′-AGC CGC CAT TGA TGG TCT CC-3′
Aggrecan (Rabbit)	F: 5′-GCT ACG GAG ACA AGG ATG AGT TC-3′ R: 5′-CGT AAA AGA CCT CAC CCT CCA T-3′
GAPDH (Rabbit)	F: 5′-TCA CCA TCT TCC AGG AGC GA-3′ R: 5′-CAC AAT GCC GAA GTG GTC GT-3′ (R)

### Animal model

#### Intraarticular injection of CM-DiI-labeled rADSC/hydrogel constructs into rabbit synovial cavities to evaluate rADSC chondrogenesis *in vivo*

Three-month-old New Zealand white rabbits were purchased from the National Laboratory Center, and the *in vivo* experiments were performed with the approval of the Kaohsiung Medical University Animal Care and Use Committee. To *in situ* trace intraarticular implanted rADSCs, rADSCs were labeled with CellTracker CM-DiI (Molecular Probes, USA) prior to being seeded into the hydrogels. CM-DiI stock was produced at a 1 mg/ml concentration in ethanol. rADSCs were labeled using 4 μL CM-DiI stock/mL of PBS for 15 min at 37 °C and at 4 °C for 15 min. After a PBS washing, CM-DiI-labeled rADSCs were encapsulated in PNIPAAm, HA-PNIPAAm-CP and HA-PNIPAAm-CL hydrogels (rADSCs/hydrogel constructs) by suspending 1 × 10^6^ cells/mL CM-DiI labeled-rADSCs in a 5% w/v PBS solution of PNIPAAm, HA-PNIPAAm-CP and HA-PNIPAAm-CL hydrogel under 4 °C. Eighteen rabbits (2.5–3 kg) were randomly allocated into 3 groups (6 rabbits/group): (1) the *PNIPAAm* group, composed of rADSCs in PNIPAAm hydrogels; (2) the *HA-PNIPAAm-CP* group, composed of rADSCs in HA-PNIPAAm-CP hydrogels; and (3) the *HA-PNIPAAm-CL* group, composed of rADSCs in HA-PNIPAAm-CL hydrogels. The hair over the medial aspect of the knee was shaved and cleaned with a depilator. Next, 300 µl of CM-DiI-labeled-rADSC/hydrogel constructs in syringe were intraarticularly injected into the rabbit knee synovial cavity under anesthesia using an intraperitoneal injection of ketamine (Ketalar^®^, Parke-Davis, Taiwan) in combination with xylazine-hydrochloride (Rompun^®^, Bayer HealthCare, Germany). After 3 weeks post-implantation, rabbits were euthanized using CO_2_ inhalation. Implanted rADSC/hydrogel constructs in rabbit knee joint cavity were harvested and fixed in 4% paraformaldehyde at 4 °C for 24 h. The chondrogenesis of the rADSC/hydrogel constructs were evaluated using a hematoxylin and eosin (H&E) stain, a Safranin-O fast green stain and an immunohistochemistry (IHC) stain. CM-DiI-labeled rADSCs in rADSC/hydrogel constructs were examined using a confocal microscope for *in situ* tracing intraarticular implanted rADSCs.

#### Hematoxylin and eosin (H&E), Safranin-O staining and immunohistochemistry (IHC) for histomorphometric analysis of neocartilage formation

After 3 weeks post-implantation, rADSC/hydrogel constructs in the rabbit synovial cavity were harvested and evaluated histomorphometrically. All specimens were fixed and paraffin-embedded as described previously^25^. The 5-μm-thick sections were stained with H&E (Santa Cruz, Santa Cruz, CA, USA). Safranin-O and fast green staining were used to evaluate the sGAG production in rADSC/hydrogel constructs *in vivo*. To evaluate sGAG production, the 5-μm-thick sections of the rADSC/hydrogel constructs harvested from rabbit knee were stained with 1% Safranin-O and counterstained with 1% fast green (Sigma, Saint Louis, MO, USA). Sections were then counterstained with 0.75% hematoxylin. IHC was performed using the ImmunoCruz Staining System (Santa Cruz Biotechnology, Inc. Dallas, Texas). Sections of rADSC/hydrogel constructs were incubated in 0.1% EDTA for 10 min at 100 °C for antigen retrieval^25,26^. After incubating with 5% BSA/PBS (Sigma, Saint Louis, MO, USA) blocking solution for 2 hr at room temperature, sections were labeled with rabbit-specific anti-type II collagen antibody (dilution 1:50; Chemicon, Temecula, CA) overnight at 4 °C in a humid chamber. After washing with PBS, sections were incubated with a biotinylated secondary antibody (Dako, Carpinteria, CA) for 1 hr and then incubated with horseradish peroxidase-conjugated streptavidin (Dako, Carpinteria, CA) for 1 hr. The reaction was developed using a 3,3′-diaminobenzidine solution containing 0.01% hydrogen peroxide, resulting in a brown color^25^. Sections were then counterstained with hematoxylin. Images at 10x, 100x and 400x were taken using a microscope equipped with a digital CCD camera (Eclipse 50i; Nikon Inc., MI, USA). For quantification, the sections of Safranin-O staining and type II collagen staining were scanned with a TissueFAXS microscope (TissueGnostics GmbH, Vienna, Austria), and followed by analyzed with the analysis software TissueQuest (TissueGnostics).

### Statistical analysis

All values are expressed as the mean ±  standard error of the mean (SEM) of at least three independent experiments. A one-way ANOVA (analysis of variance) was used to test for significant differences, and multiple comparisons were performed using Scheffe’s method. Statistical significance was set at *p* < 0.05.

## Results

### Characterization of HA-cross-linked PNIPAAm (HA-PNIPAAm-CL) by FTIR spectrometric analysis

The chemically cross-linked HA-PNIPAAm-CL was synthesized by the copolymerization method (Fig. 1A). The formation of HA-PNIPAAm-CL was confirmed by an FTIR spectrometric analysis (Fig. 1B). The FTIR spectrum of uncross-linked PNIPAAm and HA showed the characteristic peaks at 1652 cm^−1^ (A), 1540 cm^−1^ (B), 1401 cm^−1^ (C) for PNIPAAm and 1037 cm^−1^ (D) for HA correspond to the reactive amide and carboxyl groups. The FTIR spectrum of the HA-MA monomer showed characteristic peaks at 1718 cm^−1^ (E) and 1408 cm^−1^ attributed to the acrylate and carboxylate groups, respectively. The characteristic peaks for monomers, acrylate group (1718 cm^−1^), and carboxylate (1401 cm^−1^) disappeared after the successful synthesis of HA-PNIPAAm-CL. Additionally, the FTIR spectrum of HA-PNIPAAm-CL showed distinct absorptions for both HA and PNIPAAm at 1037, 1652, and 1540 cm^−1^, corresponding to hydroxyl, carbonyl and the stretching of amide groups, respectively (Fig. 1B; arrow). Increased peak intensity was observed in the synthesized polymeric HA-PNIPAAm-CL for the carbonyl groups in comparison to that of HA and HA-MA. The peak at 2854 cm^−1^ corresponds to an amide with C=O stretching, and the peak at 1619 cm^−1^ accounts for a C-O with C=O combination (Fig. 1B).

### Physicochemical and morphological properties of PNIPAAm, HA-PNIPAAm-CP and HA-PNIPAAm-CL hydrogels

**Figure 2:**
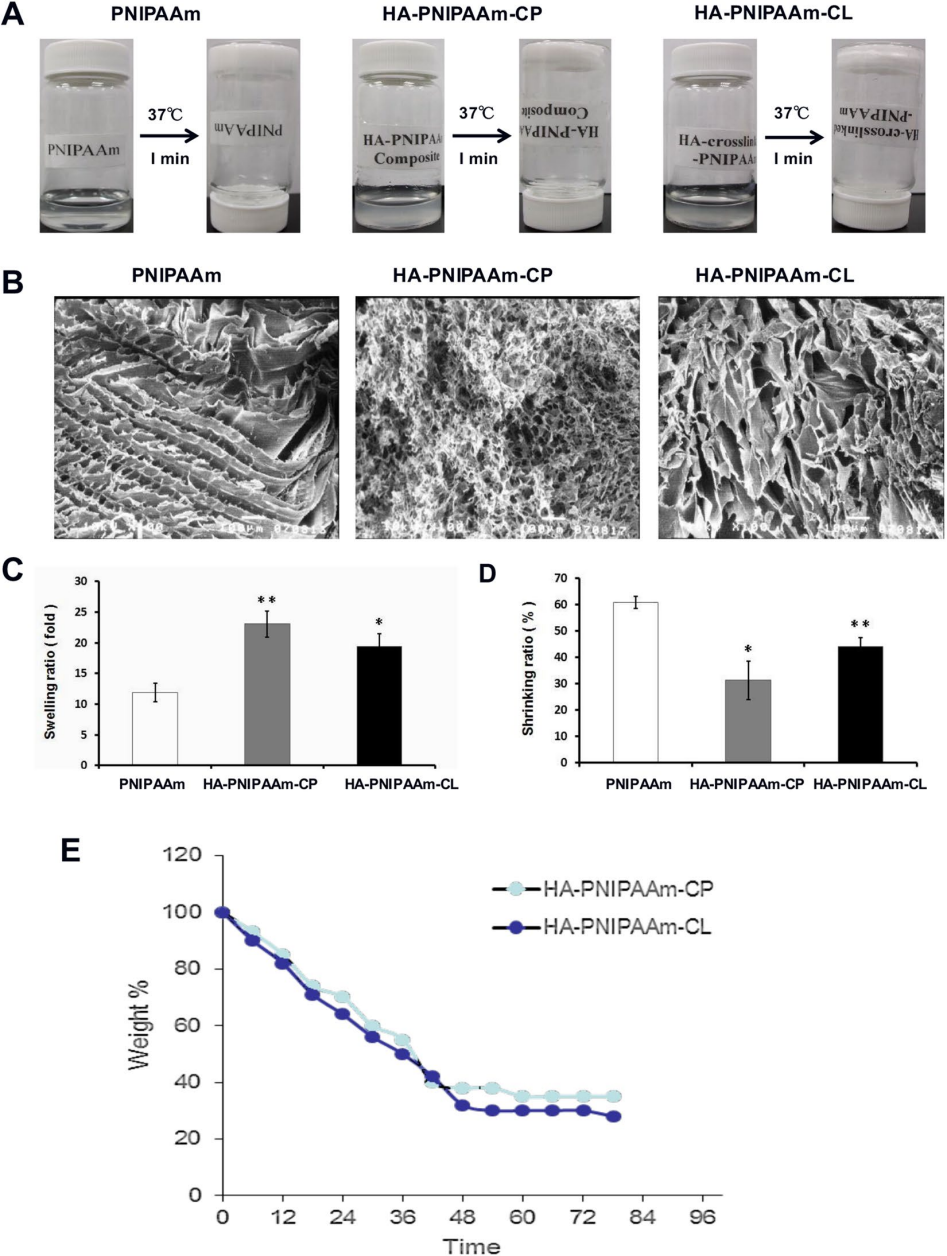
Detection of the physicochemical and morphological properties of PNIPAAm, HA-PNIPAAm-CP and HA-PNIPAAm-CL hydrogels using (**A**) LCST analysis, (**B**) SEM analysis, (**C**) a swelling test, (**D**) a shrinking test, and (**E**) *in vitro* degradation. (*) and (**) indicate p < 0.05 and p < 0.01, respectively, in comparison with the PNIPAAm group.

The hydrogelation of aqueous solutions of PNIPAAm, HA-PNIPAAm-CP and HA-PNIPAAm-CL hydrogels were measured by incubating three different concentrations (2, 5, and 10% w/v) in a water bath by increasing temperature 1 °C/min. The hydrogelation analysis showed that all hydrogels exhibit sol-to-gel phase transitions between 32 and 36 °C. The injectable 5% concentration of PNIPAAm, HA-PNIPAAm-CP and HA-PNIPAAm-CL gels showed stable hydrogel formation at 34 °C, 35 °C and 35 °C, respectively. Ten percent HA-PNIPAAm gels HA-PNIPAAm-CP and HA-PNIPAAm-CL showed sol-to-gel phase transitions at 32 °C and 33 °C, respectively and were difficult to inject. Therefore, the 5% gels were chosen for *in vitro* and *in vivo* experiments. The gelation time analysis showed that all hydrogels exhibit sol-to-gel phase transitions within 1 min at physiological temperature (37 °C) (Fig. 2A), suggesting that both the HA-modified PNIPAAm hydrogels did not have a substantial influence on the gelation property compared with PNIPAAm. Cross-sectional SEM images were obtained to characterize the microstructure morphologies of freeze-dried PNIPAAm, HA-PNIPAAm-CP and HA-PNIPAAm-CL hydrogels (Fig. 2B). The SEM images of the PNIPAAm and HA-PNIPAAm hydrogels showed a continuous and honeycomb-like porous structure with pore diameters in the range of 50–300 μm. Figure 2C shows that the swelling ratio of both freeze-dried HA-modified PNIPAAm hydrogels HA-PNIPAAm-CP (23.03 ± 2.15%) and HA-PNIPAAm-CL (18.43 ± 2.05%) hydrogels determined in PBS was significantly higher than that of PNIPAAm (11.88 ± 1.52%) hydrogels. However, the shrinkage ratio of HA-PNIPAAm-CP (31.00 ± 0.07%) and HA-PNIPAAm-CL (44 ± 0.03%) hydrogels was significantly lower than that of PNIPAAm (61.00 ± 0.02%) hydrogels (Fig. 2D). The *in vitro* degradation analysis showed that the degradation time for the HA-PNIPAAm-CP and HA-PNIPAAm-CL was approximately 48–72 hours at 37 °C (Fig. 2E).

### HA-modified PNIPAAm hydrogels enhanced biocompatibility and cell viability of rADSCs

**Figure 3:**
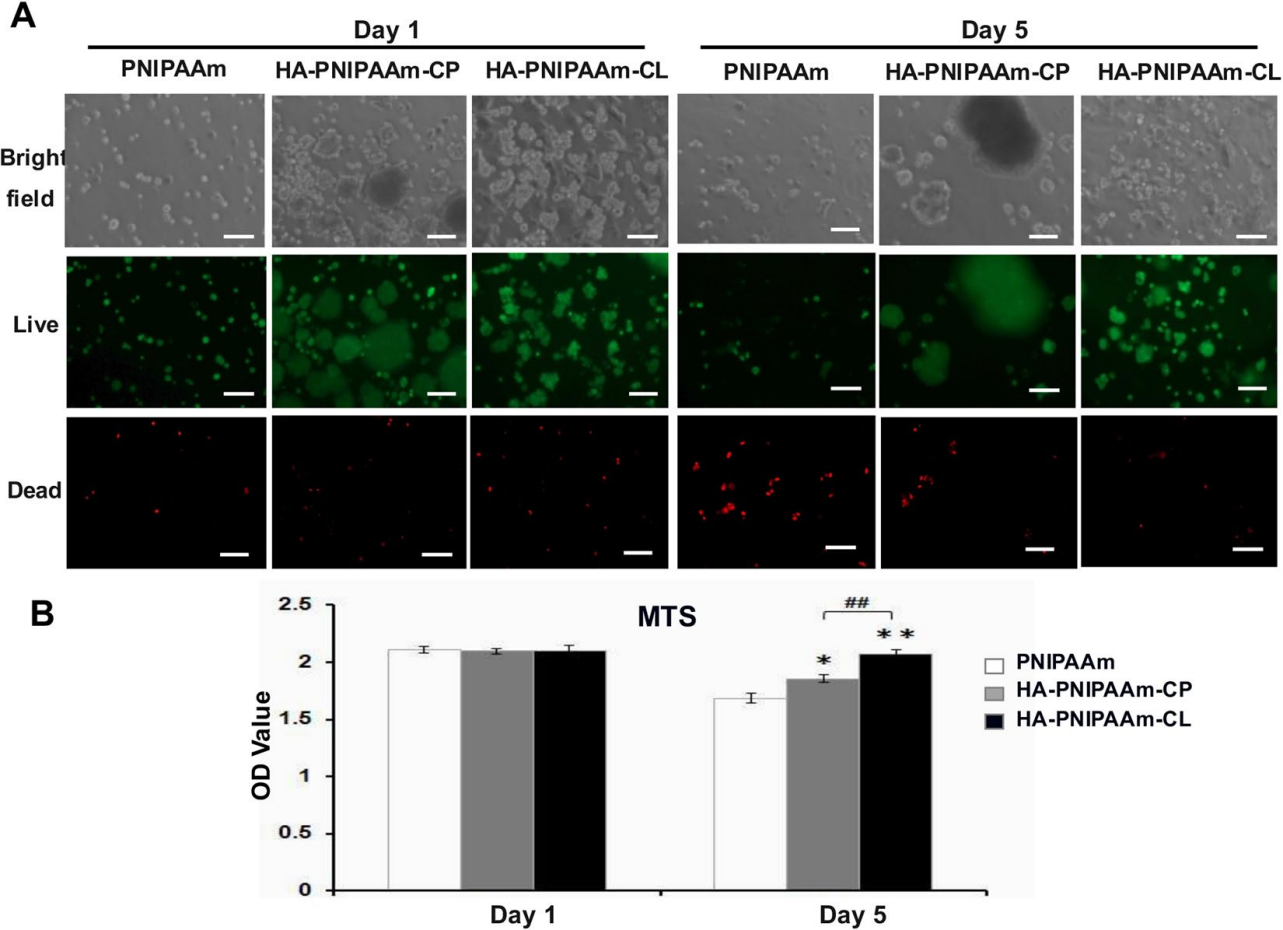
HA-modified PNIPAAm hydrogels enhanced the cytocompatibility and cell viability of rADSCs. Detection the cell survival of rADSCs encapsulated in PNIPAAm, HA-PNIPAAm-CP and HA-PNIPAAm-CL hydrogels at days 1 and 5 using (**A**) live and dead staining and (**B**) an MTS assay. Magnification: 200 X. Green: Calcein-AM. Red: EthD-1. Scale bar: 100 μm. (*) and (**) indicate p < 0.05 and p < 0.01, respectively, in comparison with the PNIPAAm group. (^##^) indicates p < 0.01 in comparison with the HA-PNIPAAm-CP group.

The live and dead staining and MTS assay were used to test the biocompatibility of PNIPAAm, HA-PNIPAAm-CP and HA-PNIPAAm-CL hydrogels. The live and dead staining images showed that higher numbers of encapsulated viable rADSCs were observed in both HA-modified PNIPAAm hydrogels than that for rADSCs cultured in PNIPAAm hydrogels at days 1 and 5 (Fig. 3A). Few dead cells were stained in all hydrogels at day 1. However, higher dead cell staining was observed in PNIPAAm hydrogels than that of HA-PNIPAAm-CP and HA-PNIPAAm-CL hydrogels at day 5; the lowest dead cell staining was displayed in HA-PNIPAAm-CL hydrogels (Fig. 3A). Cell aggregation is a critical step for initializing the process of chondrogenesis^27^. In Fig. 3A, the bright field and live staining images showed obviously aggregated cell nodules in both HA-PNIPAAm-CP and HA-PNIPAAm-CL compared to those for rADSCs cultured in PNIPAAm hydrogels at days 1 and 5. The MTS results confirmed that the cell viability significantly increased in HA-PNIPAAm-CP and HA-PNIPAAm-CL hydrogels at day 5, with the highest cell viability in HA-PNIPAAm-CL hydrogels (Fig. 3B).

### Enhancement the chondrogenic marker gene expression in rADSC-cultured HA-modified PNIPAAm hydrogels *in vitro*

**Figure 4:**
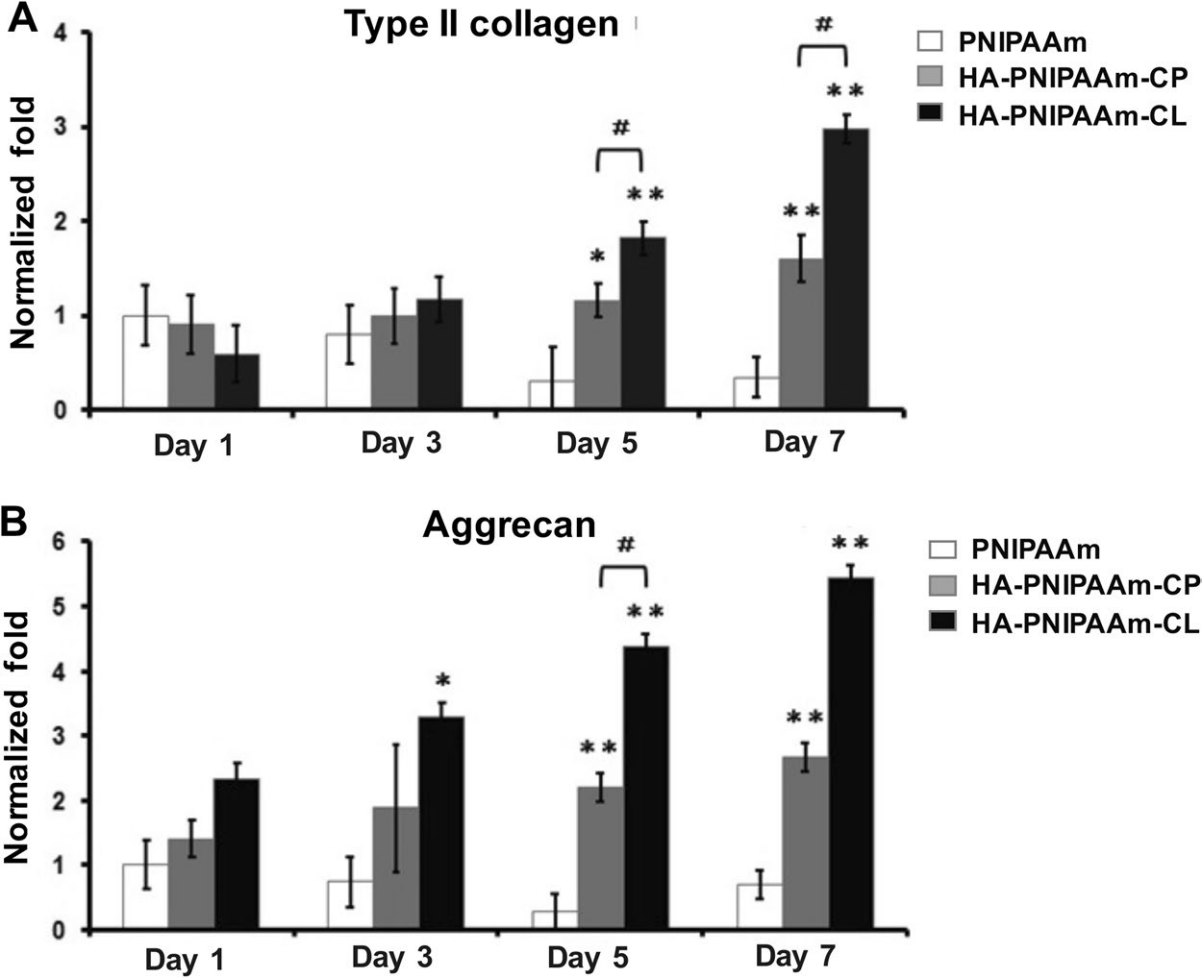
Detection the chondrogenic markers gene expression of (**A**) type II collagen and (**B**) aggrecan in rADSCs cultured in PNIPAAm, HA-PNIPAAm-CP and HA-PNIPAAm-CL hydrogels for 1, 3, 5 and 7 days. The mRNA expression level of collagen type II and aggrecan in rADSCs cultured in HA-modified hydrogels is expressed and normalized relative to the rADSCs cultured in PNIPAAm hydrogels, which is defined as 1. The values are the mean ± SEM (n = 3). (*) and (**) indicate p < 0.05 and p < 0.01, respectively, in comparison with the PNIPAAm group. (^#^) indicates p < 0.05 in comparison with the HA-PNIPAAm-CP group.

To investigate the chondro-inductive effect of HA-modified PNIPAAm hydrogels on rADSCs, quantitative real-time PCR analysis was used to measure the mRNA expression of chondrogenic marker genes, type II collagen and aggrecan, in rADSCs cultured in hydrogels for 7 days (Fig. 4). Figure 4A shows that the mRNA expression of type II collagen in rADSCs cultured HA-PNIPAAm-CP and HA-PNIPAAm-CL hydrogels increased significantly after 5 and 7 days of culturing in basal medium compared to that for rADSCs cultured in PNIPAAm hydrogels. Figure 4B shows that the mRNA expression of aggrecan in rADSCs cultured in HA-PNIPAAm-CP (at days 5 and 7) and HA-PNIPAAm-CL hydrogels (at days 3, 5 and 7) increased significantly when compared to that for rADSCs cultured in PNIPAAm hydrogels. Furthermore, the rADSCs cultured in HA-PNIPAAm-CL hydrogels showed the highest mRNA expression of type II collagen (Fig. 4A) and aggrecan (Fig. 4B) compared to that for the rADSCs cultured in HA-PNIPAAm-CP and PNIPAAm hydrogels at days 3, 5 and 7.

### Enhancement the cartilaginous matrix of sGAG production in rADSC-cultured HA-modified PNIPAAm hydrogels *in vitro*

**Figure 5:**
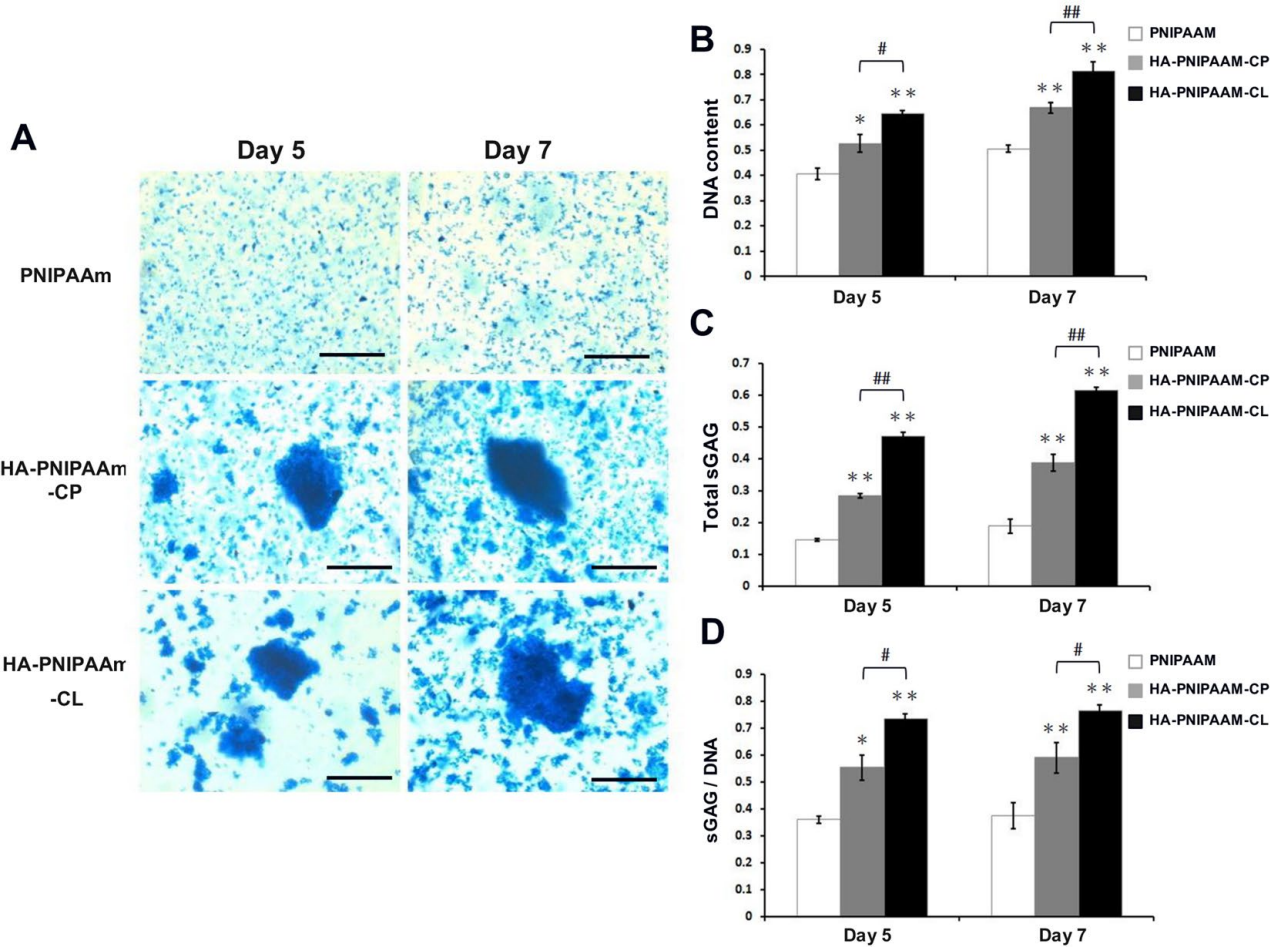
Enhancement the cell aggregation and cartilaginous matrix sGAG formation in rADSC cultured HA-modified PNIPAAm hydrogels *in vitro* at days 5 and 7. (**A**) Alcian blue staining for glycosaminoglycans (sGAG). Magnification: 400 X. Scale bar: 100 μm. (**B**) Quantification analysis of sGAG formation by using the DMMB assay. (*) and (**) indicate p < 0.05 and p < 0.01, respectively, in comparison with the PNIPAAm group. (^#^) and (^##^) indicate p < 0.05 and p < 0.01, respectively, in comparison with the HA-PNIPAAm-CP group.

The cartilaginous matrix of sGAG synthesis in the rADSC/hydrogel constructs was detected using Alcian blue staining (Fig. 5A) and quantified using DMMB assays (Fig. 5B). The Alcian blue staining results showed that higher sGAG staining and cell aggregation were observed in both HA-modified PNIPAAm hydrogel groups than that in the PNIPAAm hydrogel group at days 5 and 7 (Fig. 5A). The number of cells in the rADSC/hydrogel constructs was quantified by total DNA content (Fig. 5B). The DMMB assay showed that, at days 5 and 7, both the total amount of sGAG (Fig. 5C) and the average amount of sGAG per cell (sGAG/DNA, Fig. 5D) were significantly higher for cells cultured in both HA-modified PNIPAAm hydrogels (the highest sGAG content was in HA-PNIPAAm-CL hydrogels) than those for the cells cultured with the PNIPAAm hydrogels.

### Using rabbit model for *in vivo* evaluation of the enhancement of the neocartilage matrix formation of sGAG and type II collagen in rADSC/HA- PNIPAAm-CL constructs

**Figure 6:**
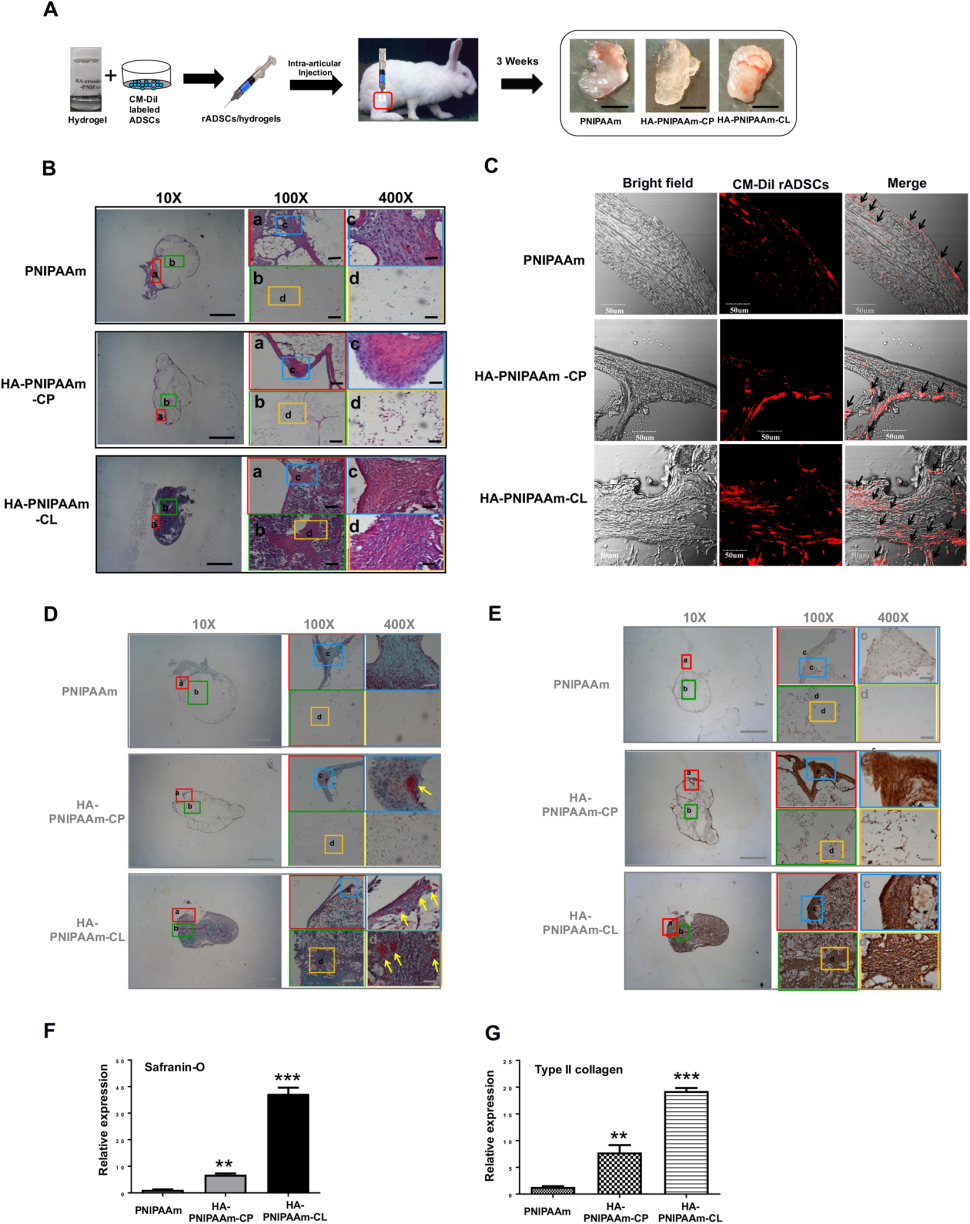
Using a rabbit model to evaluate the enhancement of the neocartilage formation in rADSCs/HA-PNIPAAm-CL constructs *in vivo*. (**A**) Schematic diagram depicting the procedure of intraarticular injection of the rADSC/hydrogel constructs into rabbit knee synovial cavities. Injected rADSC/hydrogel constructs were harvested from rabbit synovial cavities after 3 weeks and then evaluated using (**B**) H&E staining, (**C**) confocal microscopy for images of bright fields and CM-DiI-labeled rADSCs (red, arrows), (**D**) Safranin-O fast green staining to detect the deposition of sGAG (arrows), and (**E**) IHC staining for detection the type II collagen formation (brown). Bar: 50 µm. Magnification: 10X (Scale bar: 1 mm), 100X (Scale bar: 100 μm) and 400X (Scale bar: 25 μm). Quantification analysis of safranin-O staining (**F**) and type II collagen staining (**G**) normalized relative to the PNIPAAm group, which is defined as 1. (**) and (***) indicate p < 0.01 and p < 0.005, respectively, in comparison with the PNIPAAm group.

To investigate the chondroinductive effect of HA-modified PNIPAAm hydrogels on the induction of rADSC chondrogenesis *in vivo*, the rADSC/hydrogel constructs were intraarticularly injected into rabbit knee synovial cavities for 3 weeks (Fig. 6A). Injected rADSC/hydrogel constructs were gelled and formed spheroid tissue-like structures in the synovial cavities for 3 weeks (Fig. 6A) without any observed inflammation or changes in the cartilage. H&E staining of injected rADSC/hydrogel constructs (Fig. 6B) showed that tissue-like structures were observed only in the periphery of the rADSC/hydrogel constructs with the central degradation of hydrogel in the PNIPAAm and HA-PNIPAAm-CP groups. In contrast, the tissue-like structure was filled in rADSC/hydrogel constructs in HA-PNIPAAm-CL group (Fig. 6B). We tracked the implanted rADSCs in rADSC/hydrogel constructs by using CM-DiI labeling. As showed in Fig. 6C, micrographic images under confocal microscope showed the presence of implanted CM-DiI-labeled rADSCs after 3 weeks in all three groups (Fig. 6C).

Consistent with the histological results in Fig. 6B, Safranin-O staining results showed obvious deposition of sGAG in HA-PNIPAAm-CL-encapsulated rADSCs after 3 weeks of implantation (Fig. 6D). In comparison to the PNIPAAm group, sGAG deposition in HA-PNIPAAm-CL and HA-PNIPAAm-CP groups was significantly increased at 3 weeks (Fig. 6F). Moreover, the sGAG deposition was filled in the HA-PNIPAAm-CL constructs, whereas the sGAG deposition was observed only in the periphery of HA-PNIPAAm-CP constructs with no sGAG deposition in the central degradation of the hydrogel (Fig. 6D). Consistent with Fig. 6D, the quantification results of Safranin-O staining (Fig. 6F) showed that HA-PNIPAAm-CL constructs had the highest sGAG deposition. Figure 6E shows the type II collagen staining of injected rADSC/hydrogel constructs from the synovial cavity of rabbits at week 3. The HA-PNIPAAm-CL and HA-PNIPAAm-CP groups showed significantly higher type II collagen staining than the PNIPAAm group did at week 3 (Fig. 6E,G). The type II collagen deposition was filled in the HA-PNIPAAm-CL constructs, whereas the type II collagen deposition was observed mostly in the periphery of HA-PNIPAAm-CP constructs with little type II collagen depositions in the central degradation of hydrogels. The highest type II collagen deposition was found in the HA-PNIPAAm-CL constructs (Fig. 6G) These results indicated that HA-PNIPAAm-CL provides a suitable microenvironment to induce chondrogenesis in rADSCs.

## Discussion

Hyaluronic acid is a major glycosaminoglycan and native to cartilage tissue^5^. We previously showed that the HA microenvironment enhanced chondrogenesis in ADSCs^5,28^. Additionally, we found that the HA microenvironment induces chondrogenesis in hADSCs mainly through CD44 mediation^28^. In this study, we developed a two-step copolymerization method to synthesize and showed the chondroinductive property of HA-PNIPAAm-CL on rADSCs *in vitro* and *in vivo* of rabbit synovial cavity. Although a range of PNIPAAm-grafted hydrogels has been reported^11–13^, the *in vivo* effect of these hydrogels on ADSCs was not well evaluated. In this study, we demonstrated that HA-PNIPAAm-CL provides a suitable microenvironment to support the injected ADSCs in the rabbit synovial cavity successfully after 3 weeks. The *in vivo* results were consistent with our findings *in vitro* that HA-PNIPAAm-CL enhanced rADSC chondrogenesis by promoting the expression of chondrogenic marker genes (type II collagen and aggrecan) and increased cartilaginous matrix synthesis (sGAG and type II collagen) *in vivo* in rabbit synovial cavity. In addition, our study demonstrated that the synovial cavity injection model of rabbit provides an easy and effective way to evaluate the chondroinductive property of hydrogels on ADSCs *in vivo*, which can be used to screen the feasible candidate of chondroinductive hydrogels of ADSCs before testing on high-cost and time-consuming large animals.

The physical properties of the hydrogels are crucial for tissue engineering applications such as the substitution degree of methacrylated HA, degradation time and swelling/shrinkage ratio. We conducted the ^1^H-NMR analysis to confirm and measure the degree of methacrylated HA as reported previously^7^. The ^1^H-NMR analysis for the methacrylate groups confirmed that the substitution degree of methacrylated HA is ~30 mol%. To investigate whether HA-PNIPAAm-CP and HA-PNIPAAm-CL were biodegradable *in vitro*, we tested the degradation time using hyaluronidase at 37 °C. The *in vitro* degradation analysis showed that the degradation time for the HA-PNIPAAm-CP and HA-PNIPAAm-CL was 48–72 hours at 37 °C, which indicated that the HA-PNIPAAm-CP and HA-PNIPAAm-CL were biodegradable hydrogels. Since PNIPAAm is a hydrophobic polymer with high molecular interactions, such as hydrogen bonds and hydrophobic effects^10^, the incorporation of hydrophilic HA may reduce the number of hydrophobic groups and increase the hydrophilicity of the HA-PNIPAAm-CP and HA-PNIPAAm-CL hydrogels, which enhanced the swelling ratio and reduced the shrinkage ratio. Additionally, the mixing or chemical crosslinking did not affect the hydrogenation temperature of the PNIPAAm hydrogels. After gelation, PNIPAAm hydrogel shrank to a smaller size, which is not suitable for the exchange of oxygen and signaling factors in the microenvironment. To maintain the microenvironment of the stem cells within the hydrogel, the swelling ratio should be higher than that for the PNIPAAm group after gelation to facilitate the exchange of oxygen and signaling factors in synovial fluid, and the shrinkage ratio should be lower than that for the PNIPAAm group after gelation^29^. In this study, both HA-PNIPAAm-CP and HA-PNIPAAm-CL hydrogels showed a higher swelling ratio and lower shrinkage ratio than that for the PNIPAAm hydrogels (control group), which may facilitate the exchange of materials in the synovial cavity.

When attempting to fabricate the cross-linked PNIPAAm thermoresponsive hydrogel, the newly developed hydrogels should have an LCST within the physiological range^14,19^. Additionally, the gelation time plays an important role in the *in vivo* implantation. Delaying the gelation time may affect the homing of endogenous stem/progenitor cells and filling of the hydrogel at the target/defect site. The blood flow and local fluid at the target/defect site may dilute the implantation materials and affect the gelation property. In this study, the LCST analysis result demonstrated that the gelation times for HA-PNIPAAm-CP and HA-PNIPAAm-CL are within 1 min at the physiological range (37 °C). In addition, the neocartilage formation of hydrogels *in vivo* was comparable to that of the *in vitro* results; therefore, the gelation property and structural changes of hydrogels after intraarticular injection might be similar to that of *in vitro* evaluation. The reason that the different results of the degradation rate from *in vitro* and *in vivo* studies may be due to the mechanical force during the joint movement *in vivo* study. The compression and shearing forces during joint movement may cause more fluid influx and efflux into the hydrogel construct, which may cause a more rapid loss of HA mixed in HA-PNIPAAm-CP than that of HA cross-linked in HA-PNIPAAm-CL.

In this study, the histological results in the HA-PNIPAAm-CL groups showed that the cartilaginous tissue structure was observed in the whole implant, while that was only observed in the periphery of the PNIPAAm and HA-PNIPAAm-CP groups with the central degradation of hydrogel (Fig. 6B). We therefore proposed that the HA conjugated in the HA-PNIPAAm-CL may maintain in the hydrogel scaffold and provide a proper HA microenvironment for the chondrogenesis of ADSCs, whereas the HA mixed in the HA-PNIPAAm-CP can easily be dissolved out from the hydrogel due to mechanical loading in the synovial cavity, the high water solubility of HA and the simplicity of the preparation method (lyophilization of HA/PNIPAAm water solution). The cartilage formation ability of PNIPAAm and HA-PNIPAAm-CP groups is weak; they cannot provide a suitable 3-D microenvironment for cell survival and hold the cells for enough time to differentiate and form a cartilage matrix. The tissue-like structure formed at the periphery by receiving more oxygen and signal factors from the synovial fluid but not in the center of constructs. Therefore, the hydrogel in the center of the constructs in the PNIPAAm and HA-PNIPAAm-CP groups eventually degraded. In contrast, the HA-PNIPAAm-CL group has better chondrogenesis ability in that the neoformed cartilaginous matrix replaces the hydrogel both in the center and at periphery of the construct; therefore, no degradation was observed in the center of the construct.

The *in vivo* experiment of injection the rADSC/hydrogel construct in a rabbit synovial cavity is to primarily test the efficacy of cartilage formation of the construct in the synovial cavity environment, the articular cartilage’s natural environment. In addition, the animal model used in this study is to screen the feasible candidate products of hydrogels before testing on high-cost and time-consuming large animals. In this study, although it is difficult to collect the microscale cell/hydrogel constructs and rabbit synovial fluid to quantitatively evaluate the neocartilage formation and remaining HA content with time after the *in vivo* application to explain the superiority of HA-PNIPAAm-CL over HA-PNIPAAm-CP *in vivo*, our results support this and showed that the rADSCs/HA-PNIPAAm-CL group exhibited the greatest chondrogenic differentiation of ADSCs over that of the rADSCs/PNIPAAm and rADSCs/HA-PNIPAAm-CP groups *in vivo* after 3 weeks.

In stem cell therapy, regenerating articular cartilage defects and maintaining the repaired cartilage for a long time is still an unsolved problem. The survival of the transplanted/injected cells at the site of tissue damage is paramount for successful regeneration^30,31^. In this study, the PNIPAAm and the HA-PNIPAAm-CP groups show more dead cells after 5 days of culture in 3D hydrogel constructs. We proposed that the dead cells in the PNIPAAm and HA-PNIPAAm-CP groups may be due to their 3-D microenvironment, which cannot provide a suitable niche for cell survival for 5 days in basal medium. Recent reports on animal models and humans suggest that only a small percentage of stem cells remain a week after transplantation^31^. Herein, we developed an *in vivo* model that contains the exact hypoxic and physiological degradation enzymes of a chondro-specific microenvironment by *in situ* injection of rADSC/hydrogel constructs into the rabbit knee joint cavity to evaluate the chondrogenic effect of injectable HA-PNIPAAm-CL and rADSC/HA-PNIPAAm-CP hydrogels. In the present study, the cell tracking images of the rADSC/HA-PNIPAAm-CL hydrogel demonstrated the presence of injected rADSCs after 3 weeks of implantation, which suggest that HA-PNIPAAm-CL hydrogel may provide a biocompatible microenvironment to facilitate the survival of transplanted/injected ADSCs *in vivo*. However, further *in vivo* studies are required to determine whether this HA-PNIPAAm-CL hydrogel combined with rADSCs is beneficial for long-term articular cartilage regeneration in a focal defect.

In conclusion, in this study, we developed a two-step copolymerization method to synthesize HA-PNIPAAm-CL with gelation occurring under physiological temperature to create biodegradable hydrogels. We demonstrated that HA-PNIPAAm-CL enhanced rADSC chondrogenesis by promoting the expression of neoformed cartilaginous matrix synthesis of sGAG and type II collagen *in vivo* in a rabbit synovial cavity. Most importantly, the neoformed cartilaginous tissue of the HA-PNIPAAm-CL/rADSC constructs sustained in a rabbit joint cavity for 3 weeks. These results indicate that the injectable HA-PNIPAAm-CL hydrogel has high potential as an ADSC delivery biomaterial with the beneficial properties of enhancing the cytocompatibility and chondrogenesis of ADSCs to facilitate neocartilage formation *in situ* by providing a chondroinducive microenvironment for the ADSC-based tissue engineering of articular cartilage.
